# Liver Health and Dementia in an Italian Older Population: Findings From the Salus in Apulia Study

**DOI:** 10.3389/fnagi.2021.748888

**Published:** 2021-12-08

**Authors:** Luisa Lampignano, Rossella Donghia, Chiara Griseta, Gianvito Lagravinese, Sabrina Sciarra, Roberta Zupo, Fabio Castellana, Ilaria Bortone, Vito Guerra, Sarah Tirelli, Sara De Nucci, Rossella Tatoli, Madia Lozupone, Giancarlo Sborgia, Antonio Leo, Giovanni De Pergola, Gianluigi Giannelli, Francesco Panza, Rodolfo Sardone

**Affiliations:** ^1^Unit of Research Methodology and Data Sciences for Population Health, “Salus in Apulia Study” National Institute of Gastroenterology “S. de Bellis” Research Hospital, Bari, Italy; ^2^IRCCS ICS Maugeri, Bari, Italy; ^3^Department of Basic Medicine, Neuroscience, and Sense Organs, University of Bari Aldo Moro, Bari, Italy; ^4^“Santa Chiara” Institute, Lecce, Italy; ^5^Department of Biomedical Science and Human Oncology, School of Medicine, University of Bari Aldo Moro, Bari, Italy

**Keywords:** NAFLD (non-alcoholic fatty liver disease), aging, dementia, older population, neurodegenerative diseases, liver

## Abstract

**Objectives:** Non-alcoholic fatty liver disease (NAFLD) currently affects a quarter of the global population. Systemic inflammation, metabolic syndrome, and coronary artery disease, all conditions associated with NAFLD, have also been related to cognitive dysfunction in older age. The present study aimed to investigate the relationship between NAFLD risk and a dementia diagnosis in a large population-based sample aged > 65 years.

**Methods:** We selected 1,542 participants (723 men) from the Salus in Apulia Study. To assess the risk of fat distribution in the liver, we used the Fatty Liver Index (FLI). Dementia was diagnosed according to the American Psychiatric Association criteria (DSM-5).

**Results:** The overall prevalence of dementia was 8.5% [95% confidence interval (CI): 7–10%]. Subjects with dementia were older [effect size (ES): −0.89, 95% CI: −1.07 to −0.70], had a lower level of education (ES:0.88, 95% CI:0.69–1.06), higher levels of gamma-glutamyl transferase (ES: −0.21, 95% CI: −0.39 to −0.03), lower levels of total cholesterol (ES: −0.24, 95% CI: −0.42 to −0.06) and low-density lipoprotein cholesterol (ES: −0.20, 95% CI: −0.38 to 0.02), and a higher FLI (ES: −0.22, 95% CI: −0.39 to −0.04). In the logistic regression model adjusted for age, sex, education, hypertension, diabetes mellitus, alcohol consumption, smoking habits, stroke, cholesterol, and Apo-E, a dementia diagnosis was positively associated with FLI > 60 [odds ratio (OR):1.81; standard error (SE): 0.53; 95% CI: 1.02–3.21].

**Conclusion:** Our findings suggested that an increased NAFLD risk may be associated to dementia and cognitive decline in older age. Considering the high NAFLD prevalence, the possible adverse disease effects on cognitive performance pose a health problem with significant social and economic implications.

## Introduction

In Western countries, non-alcoholic fatty liver disease (NAFLD) is the most prevalent cause of chronic liver diseases (CLDs), a major cause of multimorbidity and mortality worldwide. The Third National Health and Nutrition Examination Survey (NHANES-III), conducted in the U.S. population between 1988 and 2008, showed that the prevalence of CLDs increased from 12 to 15% (Younossi et al., 2011). In Western countries, this tendency is mainly driven by the rising impact of NAFLD, and, as the populations age, the number of patients affected by this condition is expected to increase. NAFLD is common in older adults, and its prevalence rises with age, reaching 30% from the age of seventy onward (Bertolotti et al., 2014). During aging, the concomitance and increasing weight of several biomarker (i.e., metabolic syndrome, obesity, dyslipidemia, type 2diabetes) and lifestyle risk factors (i.e., Western diet pattern and sedentary habits) predisposing to NAFLD can partly explain this rising prevalence (Bertolotti et al., 2014; Younossi et al., 2021).

The rapid growth of the global population aged 65 and older has driven recent scientific interest in cognitive aging (World Health Organization [WHO], 2021). Indeed, severe cognitive impairments, such as dementia, lead to substantial cognitive dysfunction that impairs performance in daily living and global functional status activities. Dementia is defined by the WHO as a syndrome featuring the deterioration of cognitive and social functions such as memory, attention, problem-solving, and judgment (World Health Organization [WHO], 2021). Dementia has several physical, psychological and social repercussions both on people with dementia and their families, and on the whole population. A classic way to conceptualize dementia is to consider two major categories of disease: “neurodegenerative” [i.e., Alzheimer’s disease (AD)] and “non-neurodegenerative” (i.e., cerebrovascular disease). This dichotomy is a valuable heuristic, although it is constrained by its simplicity (Gale et al., 2018). Most dementia in the elderly have a neurodegenerative nature (Gale et al., 2018). Diseases can impair cognition without causing a decline in daily functioning, whether at the time of diagnosis or later. Mild neurocognitive disorder and mild cognitive impairment are two terms that have been used to describe these conditions (Gale et al., 2018).

In Europe, dementia affects approximately 10 million people, while in Italy, its prevalence ranges between 5 and 7% among older people over 65 years (Niu et al., 2017). People living with dementia lose their autonomy over time and need assistance to accomplish even the simplest activities, such as personal care. The need for ongoing care causes increased healthcare costs to enable appropriate management and care of these patients.

Starting from these concepts, prevention appears to play a central role in the optimal management of cognitive aging, recognizing potentially modifiable risk factors to reduce the risk of cognitive decline and achieving early recognition of individuals at risk of dementia to slow the progression to more severe conditions. Based on a growing body of scientific evidence, the 2017 Lancet Commission identified nine risk factors for dementia: less education, hypertension, hearing impairment, smoking, obesity, depression, physical inactivity, diabetes mellitus, sleep deprivation, and chronic isolation/loneliness (Livingston et al., 2017; Wu et al., 2019). In 2020, the Lancet Commission added excessive alcohol consumption, traumatic brain injury, and air pollution to the “life-course model” of dementia to be prevented (Livingston et al., 2020). The 12 risk factors listed above are responsible for about 40% of the world dementia cases. By modifying these both biomarkers and lifestyle risk factors, dementia could be prevented or delayed.

A link between NAFLD and brain health has been suggested (Yilmaz and Ozdogan, 2009). Liver steatosis may be linked with brain structure and function, at least partly, through shared risk factors including diabetes mellitus, obesity, and physical inactivity, which are established risk factors for dementia (Gorelick et al., 2011), as well as for brain aging (Debette et al., 2011). Interestingly, both NAFLD and dementia share two important biological risk factors, such as Apolipoprotein E (APO-E) (Yang et al., 2005; Rasmussen, 2016) and Adiponectin (ADPN) (Buechler et al., 2011; Rizzo et al., 2020). Nevertheless, although both NAFLD and the degree of liver fibrosis are linked to a wide range of adverse health outcomes, their link to cognitive performance in older people is unclear (Weinstein et al., 2019). However, in a study that compared cognitive performance in 874 NAFLD and healthy controls, the NAFLD group performed less well in activities requiring memory and attention (Seo et al., 2016). A deterioration in the cognitive performance of NAFLD subjects was also found in other smaller cross-sectional studies (Kjærgaard et al., 2021). Nevertheless, another report from the NHANES did not show specific cognitive impairment in NAFLD, and nor did other studies (Weinstein et al., 2018). Recent Italian population-based results suggest that advanced liver fibrosis could be a long-term predictor for overall dementia in people with physical frailty (Solfrizzi et al., 2020).

The present study investigated the cross-sectional relationships between a non-invasive risk score for NAFLD (fatty liver index, FLI) and a dementia diagnosis in an older population (aged over 65 years) from Southern Italy selected from the large population-based Salus in Apulia Study.

## Materials and Methods

### Study Population and Design

Participants of the present study were recruited from the electoral rolls of Castellana Grotte, Bari, Southern Italy. The sampling framework was the health registry office list until December 31, 2014, which included 19,675 subjects, 4,021 of whom were aged 65 years or more. All subjects belonged to the ‘‘Salus in Apulia Study,’’ a public health initiative funded by the Italian Ministry of Health and Apulia Regional Government and conducted at IRCCS ‘‘S. De Bellis’’ Research Hospital. From the whole sample of 4,021 subjects, only 1,542 who underwent the physical and neurological assessment, and complete blood tests, enrolled from 2015 to 2018, were included in this analysis. The subjects had the neurological and blood evaluation in the same week, contextually. The exclusion criteria were to have no complete data about neuropsychological examination. physical examination and blood biomarkers (complete case analysis). All participants signed informed consent before their examination. The Internal Review Board (IRB) approved the study of the head institution, the National Institute of Gastroenterology and Research Hospital ‘‘S. de Bellis’’ in Castellana Grotte, Italy, in 2014. The present study adhered to the ‘‘Standards for Reporting Diagnostic Accuracy Studies’’ (STARD) guidelines^1^, the ‘‘Strengthening the Reporting of Observational Studies in Epidemiology’’ (STROBE) guidelines^2^ and is in accordance with the Helsinki Declaration of 1975.

### Clinical and Socio-Demographic Characteristics

Smoking status was assessed with the single question, “Are you a current smoker?” The level of education is expressed in years of schooling. Alcohol consumption was evaluated by a validated food frequency questionnaire (FFQ) (Leoci et al., 1993). A sphygmomanometer YTON and a stethoscope FARMAC-ZARBAN were used to measure blood pressure by nurses with professional qualifications in Italy. Blood pressure was determined in a sitting position after rest. The final blood pressure values (systolic and diastolic blood pressure) were the mean of the last two of three measurements. Body mass index (BMI) was calculated as kg/m^2^. Height and weight measurements were performed using a Seca 220 stadiometer and a Seca 711 scale. Waist circumference was measured at the narrowest part of the abdomen or in the area between the tenth rib and the iliac crest (minimum circumference). Blood samples were collected from each subject in the morning after an overnight fast. Plasma glucose was determined using the glucose oxidase method (Sclavus, Siena, Italy), while the concentrations of plasma lipids [triglycerides, total cholesterol, high-density lipoprotein (HDL) cholesterol] were quantified by an automated colorimetric method (Hitachi; Boehringer Mannheim, Mannheim, Germany). Low-density lipoprotein (LDL) cholesterol was calculated by applying the Friedewald equation. Blood cell count, glutamyl oxaloacetic transaminase (GOT), glutamyl pyruvic transaminase (GPT), and gamma-glutamyl transferase (GGT) were measured using automatic enzyme procedures. The platelets count was determined using an XT-2000i hematology analyzer (Sysmex, Dasit, Cornaredo, Italy). Genomic DNA was manually purified from 4 mL of frozen blood samples by organic protein extraction and ethanol precipitation in accordance with standard methods (Kirby, 1957). The APO-E genotype was established by polymerase chain reaction (PCR) and agarose gel electrophoresis, as described in detail elsewhere (Seripa et al., 2006). Genotypes were considered according to the presence of at least one allele. For the apoE polymorphism, we reported ε2 (ε2/) and ε4 (ε4/) carriers. ε2 carriers were distinct as those participants showing at least one ε2 allele, that is, having a ε2/ε2, a ε2/ε3, or a ε2/ε4 genotype. Likewise, ε4 carriers were defined as those participants showing at least one ε4 allele, that is, having a ε4/ε2, a ε4/ε3, or a ε4/ε4 genotype (Seripa et al., 2006). We considered APO-E ε2 carriers as at decreased risk and APO-E ε4 carriers as at increased risk for late-life cognitive decline (Verghese et al., 2011).

In addition, the following pathological conditions were assessed, as described in detail elsewhere (Castellana et al., 2021). In accordance with the American College of Cardiology American Heart Association criteria, hypertension status was evaluated as values ≥ 130/80 mmHg (Whelton et al., 2018). Diabetes mellitus was diagnosed as fasting blood glucose ≥ 126 mg/dL. The presence of stroke was ascertained during a complete medical history questionnaire administered by a certified neurologist.

### Assessment of Non-alcoholic Fatty Liver Disease Risk

To assess the risk of fat distribution in the liver, Bedogni et al. (2006) developed an index, the FLI, that is accurate and easy to employ to predict NAFLD in the general population, applying BMI, waist circumference, triglycerides and GGT as routine measurements in clinical practice (Bedogni et al., 2006). An algorithm based on BMI, waist circumference, triglycerides, and GGT had an accuracy of 0.84 [95% confidence interval (CI): 0.81–0.87] in detecting NAFLD. This index ranges between 0 and 100, whereby NAFLD is ruled out with FLI < 30 and ruled in with FLI ≥ 60.

### Neurological and Neuropsychological Assessment

A standard neurological examination was carried out by a licensed neurologist, exploring perception, deambulation, cranial nerves, motor function (muscle tone, erectness of posture, and tropism), pathological gestures, sensory function, cerebellar and sphincter functions, deep tendon reflexes, and signs of diffuse cerebral distress. The Clinical Dementia Rating Scale (CDR) was administered to stage cognitive decline (Hughes et al., 1982). The diagnosis of dementia was made according to the Diagnostic and Statistical Manual of Mental Disorders, Fourth Edition criteria (DSM-4) (American Psychiatric Association [APA], 2000), as detailed elsewhere (Sardone et al., 2020). Global cognitive performance was assessed by the Mini-Mental State Examination (MMSE), a measure that includes 10 items to determine spatial and temporal orientation, attention, memory, language, and visuospatial functions (Measso et al., 1993).

### Statistical Analysis

The whole sample was subdivided according to the parameter: dementia vs. non-dementia. Normal distributions of quantitative variables were tested using the Kolmogorov-Smirnov test. Data are reported as mean ± standard deviations (M ± SD) for continuous measures and frequency and percentages (%) for all categorical variables. In order to focus on the practical differences, a statistical approach based on the effect size rather than the null hypothesis significance test (NHST) was adopted. Differences in the prevalence of exposure groups (FLI categories) and other categorical variables and their 95% CIs were calculated and used to assess essential differences in the magnitude of association, i.e., effect size (ES). Differences between continuous variables were calculated using Cohen’s *d* difference between means, Hedge’s *g* when the assumption of a similar variance was violated, and their ES using the CIs (Grissom and Kim, 2005). Three nested logistic regression multivariable models were built to estimate the odds ratio (OR) for dementia of all the principal variables. The goodness of fit of the models was assessed using pseudo-R squared (Pseudo-R2) and Akaike Information Criteria (AIC), measuring the amount of variance explained by the regression model. It ranges in (0,1), with a larger value indicating that the model explains more variance (higher value is better). In detail, its measure shows how much Logistic Regression model reduces the error vs. simply guessing the average probability of occurrence for each observation. Probability estimators were expressed as Odds Ratios (OR) and 95% CI. The multicollinearity of models was evaluated through the variance inflation factor (VIF), using the score of 2 as a cut-off for exclusion of the covariates with a high probability of convergence in the model. In addition, the load of every single covariate was assessed using standard error (se) to control for overfitting of the adjusted models. Confounders were selected among the retained factors related both to exposure (FLI categories) and to the probability of dementia, i.e., the risk factors: age, sex, education, hypertension, diabetes mellitus, alcohol consumption, smoking habit, stroke, cholesterol, and Apo-E (Cheng et al., 2012; Licher et al., 2019).

## Results

In the present study, conducted on 1,542 older individuals (53% women), the mean age of the whole sample was 73.51 ± 6.25 years. The prevalence of dementia was 8.5% (95% CI: 7–10%) People with dementia were older on average (ES: −0.89, 95% CI: −1.07 to −0.70), had a lower level of education (ES: 0.88, 95% CI: 0.69–1.06) and MMSE (ES: 3.19, 95% CI: 2.98–3.40), and a higher FLI score (ES: −0.22, 95% CI: −0.39 to −0.04). As to the metabolic biomarkers, subjects with dementia had lower levels of total cholesterol (ES: −0.24, 95% CI: −0.42 to −0.06) and LDL cholesterol (ES: −0.20, 95% CI: −0.38 to 0.02) and higher levels of GGT (ES: 0.2, 95% CI: 0.03–0.039) (Table 1).

**TABLE 1:** Sociodemographic and clinical variables in cognitively normal subjects and patients with dementia.

Variables	Dementia (*n* = 131) (8.5%)	Cognitively normal (*n* = 1411) (91.5%)	Effect size* (95% CI)
**Sociodemographic**			
Age (years)	78.85 ± 6.53	73.39 ± 6.12	0.89 (0.70–1.07)
Females	77 (9.40)	742 (90.60)	−0.06 (−0.15 to 0.03)
Males	54 (7.47)	669 (92.53)	0.06 (−0.03 to 0.15)
Education (years)	3.76 ± 3.07	7.06 ± 3.81	−0.88 (−1.06 to −0.69)
Smoking (yes)	8 (6.11)	110 (7.80)	0.44 (−0.03 to 0.06)
BMI (Kg/m^2^)	29.37 ± 4.24	28.92 ± 4.38	0.10 (−0.08 to 0.28)
Waist circumference (cm)	102.96 ± 9.85	102.93 ± 10.54	0.002 (−0.18 to 0.18)
MMSE	16.76 ± 5.01	27.10 ± 3.02	−3.19 (−3.40 to −2.98)
Low physical activity (Yes)	107 (81.68)	1,165 (82.57)	0.01 (−0.06 to 0.08)
Alcohol consumption (>20 g/die)	14 (16.28)	209 (19.05)	0.03 (−0.05 to 0.11)
**Biomarkers**			
FBG (mg/dl)	108.44 ± 31.53	105.64 ± 29.11	0.09 (−0.08 to 0.27)
Total cholesterol (mg/dl)	176.08 ± 39.48	184.95 ± 36.91	−0.24 (−0.42 to −0.06)
HDL cholesterol (mg/dl)	47.08 ± 12.99	49.35 ± 12.90	−0.18 (−0.35 to 0.003)
LDL cholesterol (mg/dl)	107.50 ± 31.71	113.83 ± 31.07	−0.20 (−0.38 to −0.02)
Triglycerides (mg/dl)	108.72 ± 52.12	104.37 ± 59.74	0.07 (−0.10 to 0.25)
GGT	41.03 ± 42.61	33.49 ± 35.56	0.21 (0.03–0.39)
AST	32.55 ± 22.37	32.15 ± 28.39	0.01 (−0.16 to 0.19)
ALT	25.44 ± 16.74	25.67 ± 20.66	−0.01 (−0.19 to 0.17)
***Apo-E genotype***			
Apo-E ε3/ε3 carriers	100 (76.09)	1,043 (73.99)	−0.02 (−0.15 to 0.11)
Apo-E ε2 carriers	20 (15.22)	173 (12.28)	−0.03 (−0.14 to 0.08)
Apo-E ε4 carriers	11 (8.70)	194 (13.73)	0.05 (−0.03 to 0.13)
***Clinical variables***			
Hypertension (Yes)	89 (67.94)	988 (70.02)	0.02 (−0.06 to 0.10)
Diabetes mellitus (Yes)	22 (16.79)	172 (12.19)	−0.05 (−0.11 to 0.02)
Stroke (Yes)	6 (7.23)	31 (3.06)	−0.04 (−0.10 to 0.01)
FLI	59.12 ± 25.43	53.81 ± 24.46	0.22 (0.04–0.39)
**FLI-Code**			
<30	21 (16.03)	276 (19.56)	0.03 (−0.03 to 0.10)
30–60	38 (29.01)	552 (39.12)	0.10 (0.02–0.18)
>60	72 (54.96)	583 (41.32)	−0.14 (−0.22 to −0.05)

The Salus in Apulia Study (n = 1,542). *Hedges’ effect size; CI, confidence interval. Data are shown as mean and standard deviation for continuous variables and as percentage (%) for proportions.

*BMI, body mass index; MMSE, Mini Mental State Examination; FBG, fasting blood glucose; HDL, high-density lipoprotein; LDL, low-density lipoprotein; GGT, γ-glutamyl transferase; AST, aspartate transaminase; ALT, alanine amino transferase, FLI Fatty Liver Index; APO-E, Apolipoprotein E.*

In the logistic regression models, a dementia diagnosis was positively associated with a FLI > 60 in the unadjusted model (Table 2). Subjects with a FLI > 60 showed almost twice the probability of dementia compared to subjects with lower FLI, ceteris paribus of the other confounders, in the fully adjusted model (OR:1.81; se:0.53; 95% CI: 1.02–3.21) (Table 1). The goodness of fit was assessed using pseudo R2, that showed an absolute good fit of the model (pseudo R2 = 0.1337); considering the small increase of the adjusted Pseudo-R2 in the models before and after the implementation of the confounders, we could be confident in that results, despite the presence of several variables. Moreover, overfitting was quite low considering the single standard errors (<1) of the model covariates (Table 1).

**TABLE 2:** Logistic regression multivariable models in cognitively normal subjects and patients with dementia.

Parameters	Unadjusted model				Partially adjusted model 1			
	OR	se (OR)	95% CI	*R* ^2^	OR	se (OR)	95% CI	*R* ^2^
				0.0101				0.0979
FLI (> 60)	1.73	0.32	1.21–2.48		1.68	0.32	1.16–2.43	
Age	–	–	–		1.89	0.14	1.63–2.19	
Sex	–	–	–		1.33	0.25	0.92–1.94	
Education	–	–	–		–	–	–	
Hypertension	–	–	–		–	–	–	
Diabetes mellitus	–	–	–		–	–	–	
Alcohol consumption	–	–	–		–	–	–	
Smoking habit	–	–	–		–	–	–	
Stroke	–	–	–		–	–	–	
Cholesterol	–	–	–		–	–	–	
Apo-E	–	–	–		–	–	–	

**Parameters**	**Partially adjusted model 2**				**Fully adjusted model**			
	**OR**	**se (OR)**	**95% CI**	***R* ^2^**	**OR**	**se (OR)**	**95% CI**	***R* ^2^**

				0.1313				0.1337
FLI (> 60)	1.79	0.42	1.12–2.85		1.81	0.53	1.02–3.21	
Age	1.74	0.17	1.44–2.11		1.66	0.20	1.31–2.12	
Sex	1.15	0.30	0.69–1.92		1.38	0.46	0.72–2.67	
Education	0.35	0.09	0.21–0.56		0.29	0.10	0.15–0.57	
Hypertension	0.72	0.19	0.44–1.20		1.04	0.35	0.54–2.00	
Diabetes mellitus	0.85	0.29	0.44–1.66		0.56	0.26	0.23–1.39	
Alcohol consumption	1.01	0.35	0.51–1.99		1.13	0.46	0.51–2.49	
Smoking habit	–	–	–		2.12	1.26	0.66–6.83	
Stroke	–	–	–		2.08	1.22	0.65–6.60	
Cholesterol	–	–	–		0.99	0.004	0.98–1.00	
Apo-E	–	–	–		0.54	0.27	0.20–1.44	

*The Salus in Apulia Study (n = 1,542). OR, odds ratio; se (OR), standard error of OR; CI, confidence interval; R^2^, pseudo R^2^; FLI, Fatty Liver Index.*

## Discussion

In the present study, we found a positive association between dementia and NAFLD risk assessed with the FLI in a large, older population-based sample. This association persisted after controlling for possible confounders such as age, sex, education, hypertension, diabetes mellitus, alcohol consumption, smoking habit, and stroke.

Although there is a wealth of evidence about the risk of a declining cognitive performance of older people with diabetes mellitus (Palta et al., 2014), studies of the cognitive performance in subjects with NAFLD are lacking. In a large cross-sectional study involving more than 4,000 American adults aged 20–59 years, NAFLD was independently associated with lower cognitive performance, independently of cerebrovascular disease (CVD) and its risk factors (Seo et al., 2016). Individuals with NAFLD and diabetes mellitus had a lower cognitive function, according to a large population-based cohort study (Weinstein et al., 2018). However, the presence of NAFLD without diabetes mellitus did not appear to be linked to poor cognitive function (Weinstein et al., 2018). Another study found that participants in the community-based Framingham Study with NAFLD had a cognitive function that was not statistically different from those without NAFLD (Weinstein et al., 2019). Insulin resistance may play a significant role in the link between NAFLD and cognitive function. Insulin resistance is widespread in people with NAFLD, diabetes mellitus, and cognitive impairment diseases, such as Alzheimer’s disease (AD) (Targher et al., 2005; Craft, 2007). A study on animal models suggested that increased insulin resistance (induced by nitrosamine) may result in non-alcoholic steatohepatitis (NASH) and AD (Tong et al., 2009). The role of metabolic disorders in the pathophysiology of AD is shown by the consistent correlations of serum-based liver function markers with cognitive performance and amyloid β (Aβ), tau protein, and other AD neurodegeneration biomarkers (Nho et al., 2019).

AD is the most prevalent cause of dementia in older people (No Authors Listed, 2021). The liver physiological role includes various processes engaged in pathways that contribute to the development and progression of AD. Both functional and structural deterioration of the liver reduces its ability to efficiently remove and degrade Aβ (Wang et al., 2017). A low hepatic expression of low-density lipoprotein receptor-related protein 1 (LRP1) and high levels of circulating Aβ have been reported in patients with CLDs. This is due to diminished Aβ clearance with low hepatic LRP1 activity (Wang et al., 2017). According to a recent epidemiological study, NAFLD, cirrhosis, CVD, or type 2 diabetes mellitus were the most common comorbidities associated with dementia caused by AD (Bassendine et al., 2020). Diet-induced hepatic insulin resistance is closely linked to NAFLD (Kumashiro et al., 2011). In a healthy condition, insulin enhances LRP1 translocation to the cell membrane in hepatocytes, favoring Aβ clearance by LRP1. Therefore, insulin resistance hampers or prevents this process, leading to increased Aβ levels (Tamaki et al., 2007). Furthermore, liver damage is correlated with Aβ deposition in the brain. Besides, NAFLD and NASH enhance the level of hematic cholesterol, particularly of 27-hydroxycholesterol, the form that can pass freely through the blood-brain barrier. This accumulation of cholesterol in the brain favors the production of Aβ in lipid rafts, contributing to the vicious circle supporting AD progression (Tamaki et al., 2007).

The AD pathogenesis is characterized by synaptic and neuronal degeneration and amyloid plaques, mostly consisting of Aβ peptide (Estrada et al., 2019). Through metabolic detoxification, the liver plays a critical role in the clearance of peripheral circulating Aβ. The absorption of Aβ into hepatocytes and subsequent excretion in the bile may be impaired in the presence of hepatic inflammation, especially in more advanced stages such as cirrhosis, resulting in greater Aβ levels in the circulation (Kanekiyo and Bu, 2014; Gehrke and Schattenberg, 2020). Moreover, failing autophagy (Nixon, 2013) predisposes to amyloidosis, which in turn sets the stage for microglial activation/phenotype change, increased CNS inflammation, and microglial toxicity, as well as an emerging tauopathy (modification of tau via excessive phosphorylation or disruption of phosphatase/kinase balance). These alterations may be due to both compensations for amyloidosis and systemic inflammation (Nixon, 2013), and thus metabolic alterations contributing to NAFLD.

Furthermore, evidence suggests that both NAFLD and dementia share two important biological risk factors, such as Apolipoprotein E (APO-E) and Adiponectin (ADPN). APO-E has been linked to a variety of diseases as well as altered lipid profiles (Nascimento et al., 2020). In particular, some studies suggest that the apoE ε4 allele is a risk factor for NAFLD pathogenesis (Yang et al., 2005). Moreover, the ε4 allele is a major genetic risk factor for late onset Alzheimer’s disease (Rasmussen, 2016). This association was first recognized in 1993 (Corder et al., 1993) and then has been validated worldwide. The APO-E polymorphism is undoubtedly the strongest genetic risk factor implicated in late-life Alzheimer’s disease (Hort et al., 2010). Even though APO-E was considered as a covariate in our models, it does not modify the effect of the association. This finding may be due to the presence of several epigenetic factors (i.e., environmental factors, lifestyle and gut microbiota) that may have influenced both NAFLD (Frank et al., 2021) and cognitive impairment in aging subjects (Morris et al., 2019) and then could have a hidden confounding effect or interplay between apoe4 and dementia.

Adiponectin (ADPN) is a pleiotropic plasma protein produced by adipose tissue with anti-diabetic, anti-atherogenic, and anti-inflammatory functions (Roy and Palaniyandi, 2021). Initially, it was considered that the principal function was to regulate metabolism. ADPN receptors were later discovered in the central nervous system as well (CNS) (Rizzo et al., 2020). Overall, ADPN appears to have neuroprotective effects through lowering inflammatory markers such as C-reactive protein (PCR), interleukin 6 (IL6), and Tumor Necrosis Factor a, based on its central and peripheral actions (TNFa). High levels of inflammatory cascade components, on the other hand, appear to suppress ADPN synthesis, implying bidirectional modulation. Furthermore, ADPN appears to have an insulin-sensitizing effect. As stated previously, the decline in insulin signaling is known to be linked to both cognitive impairment and liver diseases (Buechler et al., 2011; Rizzo et al., 2020).

In the present study, we observed lower levels of total cholesterol (TC) and LDL cholesterol and higher levels of GGT in subjects with dementia. In a meta-analysis including 23,338 participants, total cholesterol was not associated with cognitive outcomes or dementia in any analyses or in any of the extensive individual studies conducted in late-life, and the authors concluded that there is no biological association between late-life lipids and brain health, even though evidence suggests that high midlife TC increases risk of late-life AD, and may correlate with the onset of AD (Anstey et al., 2017). In the Honolulu-Asia Aging Study (HAAS) cohort, total cholesterol was reported to be consistently lower in men who developed dementia (Stewart et al., 2007). Nevertheless, there are still considerable gaps in the literature about associations between total cholesterol and late-life dementia (Peters et al., 2020). It is difficult to predict whether an isolated reading indicates cognitive decline without knowing an individual’s total cholesterol trajectory over time. It is also plausible that participants with high LDL cholesterol (and not HDL cholesterol) were lost from our cohort due to mortality or other multimorbidity associated with cardiovascular diseases. Very long-term cohort follow-ups with AD biomarkers, which the field currently lacks, will be needed to disentangle the impact of lipids on late-life brain health (Anstey et al., 2017).

GGT is a biomarker produced mostly by the liver and present in human epithelial cells. It’s an oxidative stress marker linked to an elevated risk of cardiovascular disease (Oni et al., 2020). Elevated GGT levels have also been linked with hepatic steatosis and liver cancer (Loomba et al., 2013). Since evidence suggests that smoking may also cause elevated GGT levels (Oni et al., 2020), we adjusted our analysis for smoking habits. Interestingly, several studies highlighted that baseline GGT level and GGT variability were independent predictors of incident dementia, confirming our findings (Kunutsor and Laukkanen, 2016; Lee et al., 2020). The present results could also reflect the fact that the GGT value is included in the formula for calculating the FLI.

### Strengths and Limitations

The strengths of the present study include its well-defined population-based sample, the standardized and clinically based evaluations. Moreover, this study benefits from good internal validity, as the subjects with dementia were older and had lower MMSE scores. Furthermore, in our population study, the prevalence of dementia reflects that of the European population (OECD and European Union, 2018), corroborating a good external validity of the dementia findings. Of note, the FLI has been validated to detect NAFLD in an Italian population and reflects the prevalence of the disease (Bedogni et al., 2005, 2006).

However, we must acknowledge some limitations. Firstly, the cross-sectional nature of the study did not allow us to establish causality in the relationship between NAFLD and dementia. Secondly, we cannot introduce any variables about the use of drugs (statins, NSAIDs, corticosteroids) because we don’t have access to that information. Moreover, our subjects did not have a diagnosis of AD but an overall diagnosis of dementia (unspecified) that could also include vascular forms with an entirely different etiopathogenesis from AD. In addition, we do not have any data about subjects with severe dementia (CDR greater than 1). They could not come to visit because of substantial impairment, generating a typical selection bias. Besides, we used MMSE that shown less sensitivity to mild cognitive impairment and lesser degrees of a cognitive deficit than other assessments. Furthermore, we did not use a gold standard method to confirm liver fat presence such as ultrasonography or biopsy, since the risk of having NAFLD was based on the FLI score.

## Conclusion

In conclusion, NAFLD may be a possible independent risk factor for dementia. Such a link might shed light on the contribution of NAFLD to late-life cognitive impairment and support a more effective management of older people with the disease. Particularly, if we consider NAFLD as a precursor stage of clinical diabetes, this finding could highlight an early role of metabolic alterations in the pathogenesis of dementia. This is especially important in light of the increasing prevalence of obesity and metabolic syndrome, which might signal increased NAFLD rates. In the near future, the NAFLD clinical assessment may well be included in the comprehensive geriatric assessment (CGA) with the aim of increasing the accuracy of determining a dementia risk in older age, and thus modifying the prevention trajectories in a primary care setting.

## Data Availability Statement

The raw data supporting the conclusions of this article will be made available by the authors, without undue reservation.

## Author Contributions

RS and LL: conceptualization. RD, FP, and RS: methodology. RD and VG: formal analysis. CG, GL, SS, RZ, FC, IB, ST, SD, RT, and ML: investigation. LL: writing—original draft preparation. CG, FP, and RS: writing—review and editing. GS, AL, GD, FP, and RS: supervision. RS and GG: project administration. GG: funding acquisition. All authors have read and agreed to the published version of the manuscript.

## Conflict of Interest

The authors declare that the research was conducted in the absence of any commercial or financial relationships that could be construed as a potential conflict of interest.

## Publisher’s Note

All claims expressed in this article are solely those of the authors and do not necessarily represent those of their affiliated organizations, or those of the publisher, the editors and the reviewers. Any product that may be evaluated in this article, or claim that may be made by its manufacturer, is not guaranteed or endorsed by the publisher.

## Footnotes

1
http://www.stard-statement.org/

2
https://www.strobe-statement.org/
