# Corrigendum: Liver health and dementia in an Italian older population: Findings from the Salus in Apulia study

**DOI:** 10.3389/fnagi.2023.1187003

**Published:** 2023-03-28

**Authors:** Luisa Lampignano, Rossella Donghia, Chiara Griseta, Gianvito Lagravinese, Sabrina Sciarra, Roberta Zupo, Fabio Castellana, Ilaria Bortone, Vito Guerra, Sarah Tirelli, Sara De Nucci, Rossella Tatoli, Madia Lozupone, Giancarlo Sborgia, Antonio Leo, Giovanni De Pergola, Gianluigi Giannelli, Francesco Panza, Rodolfo Sardone

**Affiliations:** ^1^National Institute of Gastroenterology - IRCCS “Saverio de Bellis”, Castellana Grotte, Italy; ^2^IRCCS ICS Maugeri, Bari, Italy; ^3^Department of Basic Medicine, Neuroscience, and Sense Organs, University of Bari Aldo Moro, Bari, Italy; ^4^“Santa Chiara” Institute, Lecce, Italy; ^5^Department of Biomedical Science and Human Oncology, School of Medicine, University of Bari Aldo Moro, Bari, Italy

**Keywords:** NAFLD (non-alcoholic fatty liver disease), aging, dementia, older population, neurodegenerative diseases, liver

In the published article, there was an error in affiliation [1]. Instead of “[Unit of Research Methodology and Data Sciences for Population Health, “Salus in Apulia Study” National Institute of Gastroenterology “S. de Bellis” Research Hospital, Bari, Italy]”, it should be “[National Institute of Gastroenterology - IRCCS “Saverio de Bellis”, Castellana Grotte, Italy]”.

The authors apologize for this error and state that this does not change the scientific conclusions of the article in any way. The original article has been updated.

